# Parliament and the Profession

**Published:** 1915-10-09

**Authors:** 


### PARLIAMENT AND THE PROFESSION.

### Red Cross Motor-Cars.

The Chancellor of the Exchequer is considering the desirability of allowing a rebate of the duty upon motor-cars, chassis, and the parts imported for ambulance and other Red Cross use. It is also interesting to observe that the question as to whether abatement of the new motor spirit duty shall be granted to medical men is to be left to the House of Commons to decide.

### The Extra Tax on Secret Remedies.

The House of Commons has agreed to postpone the operation of the increased duty on proprietary medicines until October 20. In the ordinary course of events all existing stocks of dutiable medicines would have had to be restamped by September 29, but representations having been made to the Commissioners of Customs and Excise that this was impracticable, a concession was granted.

### Nervous Cases in Military Hospitals.

In reply to a question by Mr. Touche, Mr. Tennant stated that hospital treatment is provided for soldiers suffering from nerve disturbance and loss of mental balance in the neurological sections of twenty-three military hospitals of the United Kingdom, in which uncertifiable cases of this nature amongst the rank and file are treated. This will be continued so long as accommodation is available.

### Anti-Typhoid Inoculation.

The British Union for the Abolition of Vivisection has been complaining to the War Office that posters giving official information that anti-typhoid inoculation is voluntary had been ordered to be covered up. Technically, any attempt to conceal from soldiers their rights is, of course, an irregularity—and Mr. Tennant has given his assurance that no such action has been sactioned by the War Office. But it is unfortunate that any public society should be allowed to persuade soldiers to refuse treatment which by the consent of medical authorities in all countries is essential.

### Medical Arrangements at Gallipoli.

According to a statement made by the Under-Secretary of State for War, the arrangements for dealing with the sick and wounded at Gallipoli are entirely in the hands of the Royal Army Medical Corps. Of course, the transport of the sick and wounded involves the co-operation of the naval authorities, who have the control over the working of the ships, which is exercised through a naval hospital transport officer. There are fifty ships—of which forty-nine are hospital ships—regularly engaged on this service, which it is hoped will prove an adequate number, but as an emergency measure other transports can be utilised in addition.

### Mental Hospitals Vacated for Soldiers.

According to a statement made by the Home Secretary, nine mental hospitals have been entirely vacated to provide hospital accommodation for wounded soldiers. Between 13,000 and 14,000 lunatic patients thus displaced have been accommodated in the remaining mental hospitals, with the result that most of these have at present a larger number of inmates than would be considered advisable by the Board of Control in normal times. This arrangement, if it is justifiable at all, can only be justified by the exceptional circumstances of the time, but it is satisfactory to know that the Lunacy Commissioners who have visited the mental hospitals in question speak with approval of the efforts made by the visiting committees to minimise the disadvantages of the arrangement.

### Medical Service Sub-Committees.

We wonder what was the motive underlying a rather curious question which Mr. Goldstone asked the Parliamentary representative of the National Health Insurance Commissioners. The question was "whether a paid servant of any local medical and panel committee can act as chairman of a medical service sub-committee, and whether it is also open for the secretary of an approved society, who is appointed a member of an Insurance Committee by a town or county council, to be elected the chairman of a medical service sub-committee?" In his reply, Mr. Charles Roberts pointed out that the medical benefit regulations provide that the chairman of the medical service sub-committee of an Insurance Committee shall be selected by the members of the sub-committee from those members of the Insurance Committee appointed by the county (or borough) council, and by the Commissioners, who are neither insured persons, practitioners, nor registered pharmacists. He invited Mr. Goldstone to furnish him with particulars of any specific case in which it appears that the regulations are not being complied with, with a view to making inquiries.

### Hospitals and the Spirit Tax.

We are glad to note that in the House of Commons on September 29 last the Chancellor of the Exchequer was reminded of his promise to make provision for the repayment to hospitals of the sums paid by them annually in respect of the tax on spirits. Mr. Percy Harris asked the Chancellor if he intended to introduce a clause in the Finance Bill to provide relief from spirit duty promised to hospitals; and if not, whether he would bring it in as a Vote in Supply, and whether, if the latter course were pursued, there would be any delay in the receipt of this relief by the hospitals. Mr. McKenna replied that he proposed to submit to the House a Vote in Supply at the earliest suitable opportunity. Thereupon Mr. Leif Jones who, as one of the representatives of the temperance party, opposed the proposals originally formulated by the Government, put the following question to the Chancellor: "Can the right hon. gentleman not see the propriety of basing the amount granted to hospitals on the number of patients treated rather than as a rebate on the amount of alcoholic consumption?" In answer to this Mr. McKenna said "We proceed on a different principle based on past expenditure." We agree that if there is to be any deviation from the system at first proposed the next best method would be to make a repayment equivalent to the amount of duty paid by hospitals, say, last year. But it would obviously be desirable that the amount of the grant should be altered from time to time according to the growth of any particular hospital and the consequent increase in the amount of alcohol consumed. Mr. Leif Jones's idea of basing the amount of the grant on the number of patients treated is clearly illogical, and if it were adopted would introduce unnecessary factors. For instance, under such a system the London Temperance Hospital would receive a considerable sum in respect of duty which it does not pay; this would mean that the hospital would be subsidised by the Government, whereas what hospital authorities are asking is that they should be relieved of a burden which has been shown to be imposed on them unnecessarily. This is quite a different matter. We make further reference to the question elsewhere in this issue.

